# *MTHFR* C677T polymorphisms are associated with aberrant methylation of the *IGF-2* gene in transitional cell carcinoma of the bladder

**DOI:** 10.1016/S1674-8301(12)60015-3

**Published:** 2012-03

**Authors:** Huan Cheng, Zhonglei Deng, Zengjun Wang, Wei Zhang, Jiantang Su

**Affiliations:** Department of Urology, the First Affiliated Hospital of Nanjing Medical University, Nanjing, Jiangsu 210029, China.

**Keywords:** methylenetetrahydrofolate reductase, folate, epidemiology, methylation, bladder transitional cell carcinoma, insulin-like growth factor 2 (*IGF*-*2*)

## Abstract

The purpose of this study was to determine the relationship between methylation status of the insulin-like growth factor 2 (*IGF*-*2*) gene and methylenetetrahydrofolate reductase (*MTHFR*) C677T gene polymorphisms in bladder transitional cell carcinoma tissues in a Chinese population. The polymorphisms of the folate metabolism enzyme gene *MTHFR* were studied by restrictive fragment length polymorphism (RFLP). PCR-based methods of DNA methylation analysis were used to detect the CpG island methylation status of the *IGF*-*2* gene. The association between the methylation status of the *IGF*-*2* gene and clinical characteristics, as well as *MTHFR* C677T polymorphisms, was analyzed. Aberrant hypomethylation of the *IGF*-*2* gene was found in 68.3% bladder cancer tissues and 12.4% normal bladder tissues, respectively, while hypomethylation was not detected in almost all normal bladder tissues. The hypomethylation rate of the *IGF*-*2* gene in cancer tissues was significantly higher in patients with lymph node metastasis than in those without lymph node metastasis (46.3% *vs* 17.2%, *P* = 0.018). No association was found between aberrant DNA methylation and selected factors including sex, age, tobacco smoking, alcohol consumption and green tea consumption. After adjusting for potential confounding variables the variant allele of *MTHFR* C677T was found to be associated with hypomethylation of the *IGF*-*2* gene. Compared with wildtype CC, the odds ratio was 4.33 (95% CI=1.06-10.59) for CT and 4.95 (95% CI=1.18-12.74) for TT. *MTHFR* 677 CC and CT genotypes might be one of the reasons that cause abnormal hypomethylation of the *IGF*-*2* gene, and the aberrant CpG island hypomethylation of the *IGF*-*2* gene may contribute to the genesis and progression of bladder transitional cell carcinoma.

## INTRODUCTION

Approximately 500,000 individuals in the U.S have been diagnosed with bladder cancer[1], i.e., transitional cell carcinoma transitional cell carcinoma of the bladder. It is one of the most common malignancies affecting the genitourinary tract and is characterized by multifocality and a high incidence of recurrence[2]. Although its carcinogenesis is unclear so far, the consensus is that the accumulation of multiple genetic and epigenetic alternations lead to the activation of proto-oncogenes and/or inactivation of tumor suppressor genes[3]-[5].

Aberrant DNA methylation is now recognized as an important epigenetic alteration which occurs early in human cancers[6], including bladder cancer[7]. In general, DNA methylation alterations, which play a strong role in tumorigenesis[8], are probably the most widely studied epigenetic alterations in cancer[9] and are increasingly becoming a hot subject of research. Carcinogenesis is associated with changes in this epigenetic phenomenon, including two distinct and seemingly opposing trends: global decrease in cytosine methylation (hypomethylation or unmethylation) and methylation of cytosine in CpG islands (hypermethylation)[10]. One study, performed in Spain, demonstrated that neoplasia is correlated with overall genomic hypomethylation[11]. It reported that failure to repress genes appropriately by abnormal demethylation of tissue-restricted genes or by hypomethylation of proto-oncogenes could result in the loss of tissue specificity and could promote cancer formation.

Thus, the exploration of its modulating mechanisms is expected to play important roles in the early diagnosis, treatment and prognosis of tumors. As the monocarbon unit is required for DNA, synthesis of methylation is provided by the folate metabolism pathway. Methylenetetrahydrofolate reductase (MTHFR) plays a critical role in folate metabolism, which is an important pathway for DNA methylation. Folate metabolism impaired by the genetic variants (C667T and A1298C) of *MTHFR* could change DNA methylation pattern, including promoter hypomethylation, which has been frequently observed in cancer[12]. However, little research has been done regarding this topic in transitional cell carcinoma. Therefore, his study was conducted to investigate the correlation between the methylation pattern of the proto-oncogene *IGF*-*2* in transitional cell carcinoma and the gene polymorphism of the folate metabolism enzyme, as well as their clinical characteristics.

## MATERIALS AND METHODS

### Sample information

Frozen bladder tissue and blood samples, including 125 carcinoma samples and 125 normal tissue samples, from 125 subjects were obtained from the First Affiliated Hospital of Nanjing Medical University, China. The tumor type was classified as transitional cell carcinoma by two experienced pathologists following the World Health Organization (WHO) standard. All patients had well-documented clinical histories and follow-up information. The study was approved by our institute's Human Research Ethics Committee.

### Isolation of genomic DNA from tissues

Genomic DNA was isolated from all tissues by using Promega's wizard DNA isolation kit according to the manufacturer's instructions. Bladder cancer tissue and normal bladder tissue were obtained after surgical resection and stored at -70°C. The tissues were then incubated at 55°C in homogenization buffer containing 50 mmol/L Tris (pH 8.0), 1 mmol/L EDTA, 0.5% Tween-20, and 5 mg/mL proteinase K for 3 h, and genomic DNA was isolated using Promega's DNA isolation kit.

### PCR-based methods of DNA methylation analysis

Following the manufacturer's recommendations, approximately 500 ng of the obtained DNA was digested at 37°C for 14 h with 10 units of the methylation-specific restriction endonuclease *Hpa* II (Roche Molecular Biochemicals, Mannheim, Germany), which recognizes the methylated sequence 5′-C↓CGG-3′. The digested DNA was subjected to PCR amplification with the designed primers using Primer 3 plus Software (SourceForge, Inc., Mountain View, CA,USA) encompassing the CpG clusters in exon 9 of the *IGF2* gene. The forward primer: 5′-GAAGATGCTGCTGTGCTTCC-3′, and the reverse primer: 5′-AGTGAGCAAAACTGCCGC-3′ were synthesized commercially by TIANGEN Biotechnologies (Beijing, China). Genomic PCR without *Hpa* II digestion for each sample was used as internal control. Dilutions of DNA from the digestion reaction were then used for each PCR. PCR conditions were 2 min at 94°C, followed by 27 cycles of 94°C for 30 sec, 53°C for 30 sec, and 68°C for 1 min for each primer set. The PCR products were analyzed by 2% agarose gel electrophoresis, and the amplified bands were analyzed in UV I Tech Gel Documentation system (UVI-Tech Ltd., Cambridge, United Kingdom). Undigested DNA of each sample was selected as an internal control. All normal bladder controls were set up with each batch of transitional cell carcinoma samples processed.

### Genotyping of the *MTHFR* gene by PCR-RFLP

Genomic DNA was extracted from freshly frozen blood using a QIAamp DNA Mini Kit (Qiagen, Hilden, Germany) according to the manufacturer's instructions. The extracted DNA was stored at 4°C for subsequent analysis. Genotyping of the *MTHFR* C677T was performed by PCR-RFLP. PCR amplification was performed using 5′-CAAAGGCCACCCCGAAGC-3′ and 5′-AGGACGGTGCGGTGAGAGTG-3′ (Sangon, Shanghai, China) as the forward and reverse primer pairs, respectively. Each amplification reaction was performed in a total volume of 25 µL, containing 10×PCR buffer (1.8 mmol/L MgCl_2_), 1 U *Taq* polymerase, 2.5 mmol/L of each dNTP (Tiangen, Beijing, China), 5 pmol/L of each primer and 2 µL of genomic DNA. PCR was run at 94°C for 5 min and 34 cycles at 94°C for 45 sec, 61.5°C for 40 sec and 72°C for 50 sec. This was followed by a final extension at 72°C for 7 min. Then, 5 units of *Hin*f I enzyme was added directly to the PCR products and digested at 37°C overnight. After restriction enzyme digestion of the amplified DNA, genotypes were identified by electrophoresis on 2% agarose gels and visualized with ethidium-bromide staining under ultraviolet illumination. Genotypes were scored by an experienced reader blinded to the epidemiological data and serum lipid levels. The PCR products were purified by low melting point gel electrophoresis and phenol extraction, and then the DNA sequences were analyzed in Shanghai Sangon Biological Engineering Technology & Services Co., Ltd. (Shanghai, China).

### Statistical analysis

All statistical analyses were conducted with SPSS 11.0 (Chicago, Illinois, USA) and STATA 9.2 (Stata Corp, College Station, TX, USA) statistical software. Folate intake was calculated by monitoring the subjects' daily food intake, and then the sum of all folate taken from various foods was calculated as the total folate intake with reference to the nutrition values specified in the “food compositions”. Folate intake levels were divided into four groups according to the percentile interval P25, P50 and P75, i.e. Q1, Q2, Q3 and Q4 groups from low to high, respectively. The correlation between *IGF*-*2* gene hypomethylation frequency and age, sex, smoking history, history of alcohol consumption, history of tea consumption, sites of pathological changes and TNM staging, was done by Pearson χ^2^ test. In the case of a sample size unsuitable for Pearson χ^2^ test, Fisher exact test would be used instead. Unconditional logistic regression model was carried out to analyze the correlation between *IGF*-*2* gene methylation and *MTHFR* C677T genetic polymorphism with age, gender and folate intake as potential confounding variables.

## RESULTS

### Demographic and clinical characteristics of the study participants

We obtained bladder cancer tissues and normal tissue samples from 125 patients with transitional cell carcinoma. The subjects had a median age of 62 years; 81 (64.8%) were male and 44 (35.2%) were female. Twenty-three (54.4%) of the cases had pathological bladder changes located in the bladder side wall; 68 (27.2%) cases had pathological changes in the lateral bladder wall; 34 (18.4%) cases had pathological changes at the bladder trigone area. According to the TNM staging criteria, T1, T2, T3 and T4 accounted for 16.8%, 36.8%, 40.0%, and 6.4%, respectively. Fifty-nine (47.2%) of the cases had regional lymph node metastases, while remote metastasis only occurred in one case (0.8%).

### Methylation status of the *IGF*-*2* gene and its correlation with clinical characteristics

**Fig. 1:**
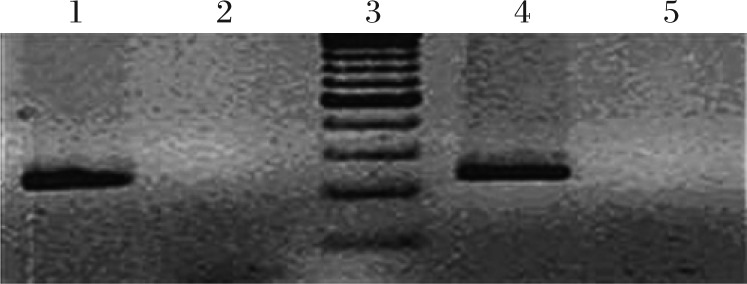
Analysis of the methylation of the *IGF2* gene by PCR-based methods. DNA samples of transitional cell carcinoma patients were studied for methylation of the *IFG2* gene by PCR-based methods as detailed in “MATERIALS AND METHODS”. Agarose gel electrophoresis showed the presence of a 214 bp DNA band in lane 1 and 4, which indicated that the CpG cluster at exon 9 of the *IGF2* gene was methylated. No such band was present at lane 2 and 5, which indicated that there was no methylation of the *IGF2* gene at exon 9 CpG cluster. lane 3: DNA 100 bp molecular weight marker.

The correlation between *IGF*-*2* gene hypomethylation frequency and age, gender, smoking, alcohol drinking, tea intake etc., was not statistically significant (***Table 1***). According to the methylation reaction electrophoresis results (***Fig. 1***), the hypomethylation frequency of the *IGF*-*2* gene in transitional cell carcinoma tissues was about 68.3% while 12.4% was detected in normal bladder tissues. The N stage in TNM staging, i.e., the occurrence of lymph node metastasis, was associated with *IGF*-*2* gene methylation frequency. Specifically, the *IGF*-*2* gene in cancer patients with lymph node metastasis had a methylation frequency of 46.3%, which was significantly lower than that in patients without lymph node metastasis (17.2%, *P* < 0.05, ***Table 2***).

**Table 1:** Association between the *IGF2* gene methylation and sex, age and selected factors in transitional cell carcinoma (TCC) patients

Variables	Cases(*n*)	Frequency of hypomethylation[n (%)]	
		TCC tissues	Normal bladder tissues
Sex			
Male	81	21(25.9)	8(9.9)
Female	44	13(29.5)	6(13.6)
* P* value		0.664	0.561
Age (years)			
<60	49	12(24.5)	8(16.3)
≥60	76	22(28.9)	6(7.9)
* P* value		0.585	0.144
Tobacco smoking			
Never	68	20(29.4)	8(11.8)
Ever	57	14(24.6)	6(10.5)
* P* value		0.544	0.827
Alcohol drinking			
Never	71	21(29.6)	8(11.3)
Ever	54	13(24.1)	6(11.1)
* P* value		0.493	0.978
Green tea drinking			
Never	77	23(29.9)	10(13.0)
Ever	48	11(22.9)	4(8.3)
* P* value		0.395	0.422

Smoking is the inhalation of the smoke of burning tobacco that is used mostly in three forms: cigarettes, pipes, and cigars. Alcohol is consumed largely for their physiological and psychological effects. The two items were divided into never and ever according to their histories. *P* values for Fisher's exact test.

**Table 2:** Association between hypomethylation of the *IGF2* and clinical characteristics in transitional cell carcinoma (TCC) patients

Variables	Cases(*n*)	Frequency of hypomethylation[n (%)]	
		TCC tissues	Normal bladder tissues
Site			
Side wall	23	8(34.8)	3(13.0)
Lateral wall	68	14(20.6)	7(10.3)
Trigone	34	12(35.3)	4(11.8)
* P* value		0.193	0.929
T stage			
Tl/2	67	18(26.9)	8(11.9)
T3/4	58	16(27.6)	6(10.3)
* P* value		0.928	0.778
N stage			
N0	66	11(17.2)	7(10.6)
N1	59	27(46.3)	7(11.9)
* P* value		0.018	0.824
M stage			
M0	124	33(26.6)	14(11.3)
M1	1	1 (100)	0
* P* value		0.272	1.000

*P* values for Fisher's exact test.

### Relationship between *IGF-2* gene methylation status and polymorphism of *MTHFR* C677T

**Fig. 2:**
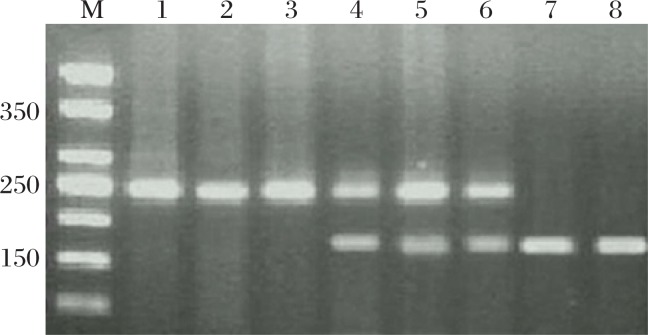
*MTHFR* C 677T gene fragment length polymorphism distribution by *Hin*f I restriction enzyme (2.5% agarose gel electrophoresis). M: 100 bp ladder Marker; Lanes 1-3: CC genotype; Lanes 4-6: CT genotype; Lanes 7 and 8: TT genotype.

We compared the correlation between the *IGF*-*2* gene methylation status and *MTHFR* genotype CC, CT and TT in transitional cell carcinoma tissues and normal bladder tissue. After adjusting other potential confounding variables such as age, gender, folate intake, we found that the *IGF*-*2* gene hypomethylation frequency in the transitional cell carcinoma tissues from carriers of *MTHFR* variant CT and TT genotypes was significantly increased compared with the *MTHFR* wildtype genotype CC, with the OR values of 4.33 (95% CI=1.06-10.59) and 4.95 (95% CI=1.18-12.74), respectively (***Figs. 2******,*** ***3*** and ***Table 3***).

**Fig. 3:**
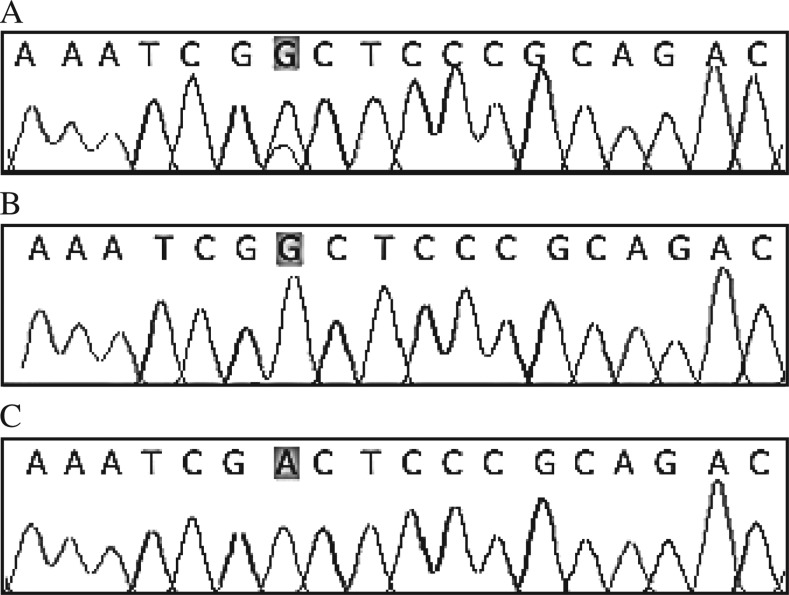
Partial nucleotide sequence of the *MTHFR* C677T locus. A: CT genotype; B: CC genotype; C: TT genotype.

**Table 3:** Association between hypomethylation of *IGF*-*2* and *MTHFR* C677T polymorphisms in transitional cell carcinoma (TCC) patients

Variables	OR(95% CI) *	
	TCC tissues	Normal bladder tissues
Age(years)	1.01(0.10-1.08)	0.91(0.83-1.00)
Sex		
Male	1.00	1.00
Female	1.32(0.53-3.24)	2.37(0.65-8.65)
Folate intake(µg/d)		
Q1	1.00	1.00
Q2	2.53(0.89-7.14)	2.65(0.58-11.99)
Q3	0.58(0.15-2.21)	0.34(0.03-3.78)
Q4	5.09(1.02-25.36)	4.23(0.51-34.79)
MTHFR C677T		
CC	1.00	1.00
CT	4.33 (1.06-10.59)	2.90(0.63-13.33)
TT	4.95 (1.18-12.74)	0.80(0.11-5.72)

*Adjusted by age, sex, folate intake and *MTHFR* C677T genotypes. The cut point of folate intake was quartile (µg/d), Ql: 0-26.2; Q2: 26.3-99.4; Q3: 99.5-311.0; Q4: ≥ 311.1.

## DISCUSSION

Genomic DNA methylation is the best-studied epigenetic modification currently discovered. The process is promoted by DNA methyltransferases which use *S*-adenosyl-methionine as the methyl donor[13]. 5-methylcytosine is formed through the addition of a methyl group to the fifth carbon position of the cytosinepyrimidine ring by DNA methyltransferases[14]. DNA methylation is an epigenetic mechanism, which plays an important role in regulating gene expression at the transcription level[15]. Through methylation of cytosine of the CpG island in the gene promoter and other CpG-riched regions, some redundant genes in certain tissues or cells can be silenced, while demethylation can function as an activator in some tissueor stage-specific genes, which contribute to the temporal and spatial regulation of gene expression[16],[17]. Previous studies have described global genomic hypomehtylation in several malignant cancers, including bladder carcinoma[18] and other organic carcinomas, by evaluating genomic repetitive sequences. Therefore, these epigenetic mechanisms in the transcriptional regulation of genes play critical roles in cancer progression[19].

IGF-2 is a member of the IGF family, which is a circulating peptide hormone and locally acting growth factor with both paracrine and autocrine functions[20],[21]. IGF-2 could encourage cell division in a variety of tissues. The *IGF*-*2* gene is located at chromosome 11p15.5 and is flanked by the *insulin* and *H19* genes in a region that is known to have differential parental methylation. The *IGF*-*2* gene is associated with CpG regions that have allele-specific DNA methylation known as differentially methylated regions (DMRs). *IGF*-*2* consists of nine exons in humans; a few are non-coding leader exons, while exons 6-9 encode prepro-*IGF*-*2* polypeptide. There are four CpG islands within the *IGF2* gene. The first CpG island maps between the first two untranslated exons and is found to be fully methylated on both alleles in all tissues. The second and third CpG islands (DMR0) map to promoter 2, 4 (P2-P4) and the IGF2 antisense (IGF2-AS) transcripts, and this region is fully unmethylated in all tissues. However, a subsequent study showed that paternal DMR0 was fully methylated, contradicting the previous studies[22],[23]. The final CpG island maps to exon 9, which has previously been defined as DMR in both human and mouse, with the paternal allele being more methylated than the maternal allele[24]. Although the *IGF*-*2* is the first autosomal gene identified to exhibit imprinting, a study indicated that 2/9 (22.2%) of cases displaying loss of imprinting (LOI) of the *IGF*-*2* and 2/16 (12.5%) had LOI of *H19* in bladder cancer[25], However, the precise regulatory mechanism of the *IGF*-*2* in transitional cell carcinoma is not clear. The present study is the first to assess the methylation status of the exon 9 CpG cluster in samples from transitional cell carcinoma and neighboring normal bladder tissue by PCR-based methylation assay. In our present study, the *IGF*-*2* gene hypomethylation rate in transitional cell carcinoma was 68.3% compared with the normal healthy controls. The presence of regional lymph node metastasis was also associated with decreased *IGF*-*2* gene methylation frequency, i.e., in the cancer tissues from patients with lymph node metastasis, the *IGF*-*2* gene hypomethylation frequency (46.3%) was significantly higher than that of patients without lymph node metastasis (17.2%), suggesting that abnormal *IGF*-*2* gene hypomethylation might serve as a valuable biomarker in transitional cell carcinoma diagnosis and a potential indicator of transitional cell carcinoma prognosis.

Unlike genetic changes, epigenetic alterations are more dynamic and often reversible, depending on the presence or removal of the inducing factors[26]. Further exploration of the impact factors for epigenetic alterations was of great value for the early prevention and intervention of diseases, but knowledge in this field is still relatively lacking[27]. It is found in this study that environmental factors (for example, dietary nutrients) are associated with genomic DNA methylation, especially deficiency in methyl group donors such as vitamin A and methionine. Zhang *et al*.[28] showed that a dietary pattern characterized by a high intake of vegetables and fruits might protect against global DNA hypomethylation. Folate is an important dietary nutrient, which accepts one-carbon units from one-carbon unit donors. DNA methylation status was determined as a functional endpoint, suggesting that abnormal folate metabolism may affect the genomic methylation state negatively. MTHFR is a key enzyme regulating folate metabolism, which affects DNA methylation and synthesis[29]. *MTHFR* converts 5, 10-methylentetrahydrofolate to 5-methyltetrahydrofolate, which is required for homocysteine methylation to methionine. Methionine is then activated to *S*-adenosylmethionine, a universal methyl donor in numerous transmethylation reactions, including methylation of DNA, RNA, proteins, and other molecules[30]. Moreover, the enzyme activities of *MTHFR* 677TT are only 30% of those of the wildtype 677CC genotypes, while the enzyme activities of the *MTHFR* 677CT are 60% of the wildtype enzyme[31]. Changing activity of *MTHFR* enzyme is associated with polymorphism in the *MTHFR* gene, which would affect its abilities involved in DNA synthesis and the supply of methyl group. Therefore, the association between *MTHFR* gene polymorphism and DNA methylation is given high priority all over the world. Friso *et al*.[29] reported that in human lymphocytes, the gene-nutrient interaction affecting DNA methylation in 1298AA was mainly due to the coexistence of the 677TT genotype. Axume *et al*.[32] suggested that the *MTHFR* 677TT genotype and folate interacted to lower global leukocyte DNA methylation patterns in young Mexican American women. Moreover, Supic *et al*.[33] found a significant association between TT genotype and methylation status of the *RASSF1A* gene in oral squamous cell carcinoma patients.

In this study, we found that carriers of *MTHFR* CC and CT mutation genotypes increased the *IGF*-*2* gene hypomethylation frequency in their transitional cell carcinoma tissues, which was associated with the level of folate intake. Source of dietary folate, amount of folate intake, as well as body metabolism will affect the supply of methyl group required for DNA methylation. Ma *et al*.[34] also provided support for an important role of folate metabolism in colon carcinogenesis. These results suggest that the 677TT mutation in *MTHFR* reduces colon cancer risk, perhaps by increasing 5,10-methylenetetrahydrofolate levels for DNA synthesis, but that low folate intake or high alcohol consumption may negate some of the protective effect. It is generally believed that with sufficient folate intake, the risk of cancer in *MTHFR* 677CT or TT genotype carriers would be reduced, since low activity of folate metabolic enzyme will facilitate DNA synthesis, while adequate folate can provide sufficient methyl groups for DNA methylation. Conversely, if folate intake is deficient, DNA synthesis, repair and methylation would all be affected. A deficiency in 5, 10-methylene THF supply would result in difficulty executing the protective function of *MTHFR* mutation genotype, therefore disturbing the DNA methylation process[35],[36]. However, during carcinogenesis, the whole genome is in a hypomethylated state, accompanied by hypermethylation of specific genes. Therefore, further studies to investigate how individual folate intake level and folate metabolism pathway disorder affect this co-existence of hyper- and hypo-methylation state seem justified.

In summary, abnormal methylation in exon 9 of the *IGF*-*2* gene might contribute to the initiation and development of transitional cell carcinoma. Individuals with *MTHFR* 677CT or TT genotype may be a positive factor in carcinogenesis by demethylating exon 9 of the *IGF*-*2* gene *in vivo*. Therefore, exploration of abnormal DNA methylation distribution is expected to play an important role in early individual diagnosis and in monitoring the prognosis of transitional cell carcinoma.
